# Supplementary material for: Dynamic dichotomy of accumbal population activity underlies cocaine sensitization
Source: eLife. 2021 Oct 5;10:e66048. doi: 10.7554/eLife.66048 (PMC8523149; doi:10.7554/eLife.66048)
Supplement: Transparent reporting form [file elife-66048-transrepform1.docx]

***eLife’s* transparent reporting form**

We encourage authors to provide detailed information *within their submission* to facilitate the interpretation and replication of experiments. Authors can upload supporting documentation to indicate the use of appropriate reporting guidelines for health-related research (see [EQUATOR Network](http://www.equator-network.org/%20)), life science research (see the [BioSharing Information Resource](https://biosharing.org/)), or the [ARRIVE guidelines](http://www.plosbiology.org/article/info:doi/10.1371/journal.pbio.1000412) for reporting work involving animal research. Where applicable, authors should refer to any relevant reporting standards documents in this form.

If you have any questions, please consult our Journal Policies and/or contact us: [editorial@elifesciences.org](mailto:editorial@elifesciences.org).

**Sample-size estimation**

- You should state whether an appropriate sample size was computed when the study was being designed
- You should state the statistical method of sample size computation and any required assumptions
- If no explicit power analysis was used, you should describe how you decided what sample (replicate) size (number) to use

Please outline where this information can be found within the submission (e.g., sections or figure legends), or explain why this information doesn’t apply to your submission:

This information is also present in the submission in the methods section under Statistical tests and experimental considerations:

No formal sample size statistics were done before the start of the project, as there was no pre-specified effect size. Rather sample sizes were estimated based on similar studies of epifluorescence miniaturized microscopy recordings in the dorsal striatum (Klaus et al <https://doi.org/10.1016/j.neuron.2017.08.015>, Barbera et al <https://doi.org/10.1016/j.neuron.2016.08.037> and Parker et al <https://doi.org/10.1038/s41586-018-0090-6>).

**Replicates**

- You should report how often each experiment was performed
- You should include a definition of biological versus technical replication
- The data obtained should be provided and sufficient information should be provided to indicate the number of independent biological and/or technical replicates
- If you encountered any outliers, you should describe how these were handled
- Criteria for exclusion/inclusion of data should be clearly stated
- High-throughput sequence data should be uploaded before submission, with a private link for reviewers provided (these are available from both GEO and ArrayExpress)

Please outline where this information can be found within the submission (e.g., sections or figure legends), or explain why this information doesn’t apply to your submission:

The following information can also be found in the methods section:

For all miniscope recordings:

Due to the difficulty of the technical preparation, there was variability in the amount of neurons recorded per animal. Animals with <20 neurons were typically excluded from the study. Due to the time-intensiveness of the recordings animals, cohorts of 2-4 animals were recorded on the same recording day. Thus for all experiments around 10-15 different “cohorts” were combined.

For fiber photometry recordings:

Cohorts of 2-4 animals were recorded on the same recording day, resulting in 3 cohorts of biological replicates.

**Statistical reporting**

- Statistical analysis methods should be described and justified
- Raw data should be presented in figures whenever informative to do so (typically when N per group is less than 10)
- For each experiment, you should identify the statistical tests used, exact values of N, definitions of center, methods of multiple test correction, and dispersion and precision measures (e.g., mean, median, SD, SEM, confidence intervals; and, for the major substantive results, a measure of effect size (e.g., Pearson's r, Cohen's d)
- Report exact p-values wherever possible alongside the summary statistics and 95% confidence intervals. These should be reported for all key questions and not only when the p-value is less than 0.05.

Please outline where this information can be found within the submission (e.g., sections or figure legends), or explain why this information doesn’t apply to your submission:

Statistical methods are described in the methods section, and specific chosen statistical tests are described in the figure legends.

Data is plotted per individual data point for all per-animal data (Ns around 8-14).

For each figure that uses statistics the statistical tests, as well as N, and where appropriate the distribution of center + spread is reported in the legends.

Where possible the exact p values are given in the figure legends.

(For large datasets, or papers with a very large number of statistical tests, you may upload a single table file with tests, Ns, etc., with reference to sections in the manuscript.)

**Group allocation**

- Indicate how samples were allocated into experimental groups (in the case of clinical studies, please specify allocation to treatment method); if randomization was used, please also state if restricted randomization was applied
- Indicate if masking was used during group allocation, data collection and/or data analysis

Please outline where this information can be found within the submission (e.g., sections or figure legends), or explain why this information doesn’t apply to your submission:

This information can also be found in the “Statistical tests and experimental considerations” part of the methods.

Animals were randomly assigned to control/test group. Neuronal identification was done automatically, curation of neuronal data was done manually but without knowledge of results. No formal blinding was done at any part of the data acquisition/analysis.

**Additional data files (“source data”)**

- We encourage you to upload relevant additional data files, such as numerical data that are represented as a graph in a figure, or as a summary table
- Where provided, these should be in the most useful format, and they can be uploaded as “Source data” files linked to a main figure or table
- Include model definition files including the full list of parameters used
- Include code used for data analysis (e.g., R, MatLab)
- Avoid stating that data files are “available upon request”

Please indicate the figures or tables for which source data files have been provided:

Data and code will be made available through an open/FAIR repository: DOI: 10.5281/zenodo.4471283
